# Improving the National Intellectual Disability Higher Trainee Experience

**DOI:** 10.1192/bjo.2025.10311

**Published:** 2025-06-20

**Authors:** Abigail Swift

**Affiliations:** Aneurin Bevan, Cardiff, United Kingdom

## Abstract

**Aims:** At several regional representative meetings held by the National Trainee representatives for the Intellectual Disability (ID) faculty within the Royal College of Psychiatrists a number of themes emerged. There were concerns around the ability to disseminate information to new starters as often groups like the Trainee ID whatsapp group are dependent upon word of mouth. Some trainees talked about difficulties meeting particular competencies such as inpatient experience whilst others felt isolated due to small training numbers. There were also changes to the portfolio system that had raised concerns for some.

Due to the small number of trainees who attend the meetings, we were unclear as to how widespread the themes were. We agreed a national survey of ID trainees would be beneficial.

**Methods:** We constructed a survey for ID and dual trainees via Google forms to assess their knowledge of the ID faculty, whether they had access to the communication channels used by trainees, trainee competencies and trainee loneliness. We advertised this survey via the trainee ID whatsapp group, through regional representatives, discussions with local training programme director (TPD) to encourage ID trainees to participate. We added a section around thoughts as to how we could improve the ID trainee experience like a virtual event and/or an induction page on the Royal College website.

**Results:** 46 people responded ranging from ST1 to ST8 across the nationals. 60.9% of ID trainees didn’t have a good understanding of the ID Faculty’s role. 80.4% didn’t feel well inducted into the portfolio. 69.5% of trainees felt sometimes or often lonely within their jobs. 50% were signed up to the Faculty Newsletter and 43.5% weren’t confidently aware of the other channels to disseminate information. There were about 33% of trainees felt they were struggling to gain experience within inpatient or CAMHS ID or forensic ID. 67.4% ID trainees supported a virtual event and 69.6% supported an induction page.

**Conclusion:** Based on the results, we proposed a virtual ‘Welcome Event’ aimed at new ID higher trainees. This was supported by the wider ID faculty. The event was held virtually on 15 October and 39 participants joined which included ID trainees and TPDs. A feedback survey was circulated to evaluate the event, unfortunately only 5 people responded. All responded positively and agreed that they would recommend this event to others. We hope this event will become a bi-annual event to help strengthen the trainee experience along with developing an induction page.

